# Ewing Sarcoma/Primitive Neuroectodermal Tumor Involving the Duodenum

**DOI:** 10.31486/toj.21.0040

**Published:** 2022

**Authors:** Dawood A. Tafti, Ilsup Yoon, Jesse Fitzgerald, Adam Graeber, Paul Clark

**Affiliations:** ^1^ Department of Radiology, Brooke Army Medical Center, Fort Sam Houston, San Antonio, TX; ^2^ Department of Pathology, Brooke Army Medical Center, Fort Sam Houston, San Antonio, TX

**Keywords:** *Abdominal pain*, *duodenum*, *neuroectodermal tumors–primitive*, *sarcoma–Ewing*

## Abstract

**Background:** Ewing sarcoma/primitive neuroectodermal tumor (ES/PNET) is a soft tissue malignancy arising from the neuroectoderm. While the locations of these extraskeletal manifestations are diverse, origin from the small bowel and small bowel mesentery is extremely rare. Intra-abdominal manifestations of ES/PNETs are nonspecific, and patients present with a wide range of symptoms, most frequently vague abdominal pain.

**Case Report:** A 66-year-old female initially presented with vague and nonspecific symptoms of hypotension, anemia, dyspnea, and coffee-ground emesis. Imaging workup with computed tomography and fluorodeoxyglucose positron emission tomography demonstrated a metabolically active large mass involving the duodenum and measuring 10.3 × 8.8 × 12.3 cm. The mass was characterized as an ES/PNET on histopathologic diagnosis. The patient was treated with chemotherapy followed by radical resection and was disease-free at 1 year postpresentation.

**Conclusion:** This case highlights that while ES/PNETs are rare tumors of the abdomen, they should be considered in cases of large soft tissue masses in patients presenting with nonspecific symptoms. To the best of our knowledge, this case is the fourth report in the literature of an ES/PNET involving the duodenum.

## INTRODUCTION

Initially described by Stout in 1918, Ewing sarcoma/primitive neuroectodermal tumor (ES/PNET) is a soft tissue malignancy arising from the neuroectoderm.^1^ These tumors typically arise from the diaphyseal and metadiaphyseal portions of long bones, ribs, and pelvis. When manifested in the musculoskeletal system, these tumors present with locoregional pain and a palpable mass. Advanced disease can also present with moderate fever, often accompanied by night sweats, and pathological fracture.^2^ Histopathologically, these tumors are composed of small round cells in a uniform distribution. These cells contain round nucleoli with fine stippled chromatin. The cytoplasm is scant, clear, or eosinophilic. Atypical features also include prominent nucleoli and irregular contours.^3^

Extraskeletal ES/PNETs occur in approximately 12% of patients with ES/PNET.^4^ While the locations of these extraskeletal manifestations are diverse, origin from the small bowel and small bowel mesentery is extremely rare. Intra-abdominal manifestations of ES/PNETs are nonspecific, and patients present with a wide range of symptoms, most frequently vague abdominal pain.^5^ We present the case of a 66-year-old female who presented with an ES/PNET involving the duodenum.

## CASE REPORT

A 66-year-old female initially presented to the emergency department (ED) for evaluation of hypotension, palpitations, and progressive dyspnea with exertion. The patient also reported palpitations and lightheadedness with positional changes for a period of 3 weeks prior to presentation, as well as a 6-month history of loss of appetite. Her medical history was notable for prediabetes, vitamin D deficiency, hyperlipidemia, and hypertension. Her surgical history was notable for cesarean section and tubal ligation. Patient's weight at presentation was 53.9 kg, and her body mass index was 22 kg/m^2^. At the time of initial presentation, the patient's medications included ramipril, metformin, and vitamin D supplement. Physical examination was notable for a small external hemorrhoid but was otherwise normal. Laboratory analysis at the initial ED visit demonstrated profound anemia with hemoglobin of 5.4 g/dL (reference range, 11.6-15 g/dL) and hematocrit of 18.3% (reference range, 35.5%-44.9%). The patient was admitted, managed with 2 blood transfusions (900 mL total), and discharged home in stable condition the next day with a consultation placed for gastroenterology.

**Figure 1.**
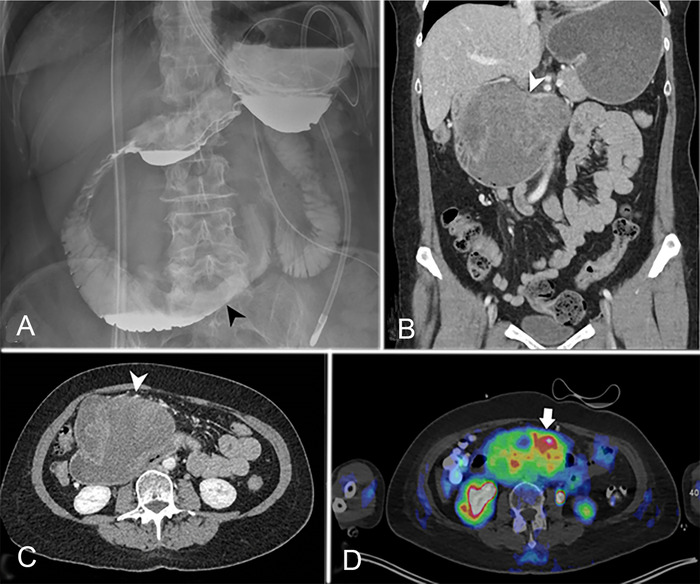
(A) Anteroposterior view of the abdomen after administration of oral contrast (Omnipaque, GE Healthcare) demonstrates narrowed third and fourth segments of the duodenum secondary to mass effect (black arrowhead). (B) Coronal and (C) axial computed tomography (CT) images demonstrate a heterogeneous mass arising from the superior and anterior walls of the third segment of the duodenum (white arrowheads). Approximately 90 mL of Visipaque 320 (GE Healthcare) was administered intravenously. (D) Fluorodeoxyglucose (F 18-FDG) positron emission tomography/CT demonstrates the same mass with intense metabolic activity (white arrow). Approximately 7.15 mCi of F 18-FDG was administered intravenously. The patient's blood glucose level just prior to imaging was 90 mg/dL. Time from F 18-FDG injection to scan was 51 minutes.

The patient returned to the ED 1 week later, presenting with new symptoms of coffee-ground emesis and abdominal pain. During the second ED visit, computed tomography (CT) of the abdomen showed a large heterogeneous mass measuring 10.3 × 8.8 × 12.3 cm (anteroposterior × transverse × craniocaudal dimensions) arising from the third segment of the duodenum (Figures 1A, 1B, and 1C). Evidence of duodenal and gastric outlet obstructions, as well as extrinsic mass effect on the pancreatic head, was also noted. No lymphadenopathy was identified. Evaluation of the mass with positron emission tomography demonstrated a maximum standardized uptake value of 6.8 without additional sites of disease (Figure 1D).

CT-guided biopsy demonstrated a malignant round cell tumor with positivity for CD99, synaptophysin, CD56, and CD117, with negative expression of CD45, CD43, pancytokeratin, beta-catenin, chromogranin, WT1, desmin, S100, DOG1, and TLE1. Fluorescence in situ hybridization (FISH) analysis performed using DNA probes for EWSR1 demonstrated an unusual variant pattern of rearrangement of EWSR1 locus at 22q12 in 52% of nuclei, with 1 fusion and 1 free 5′ EWSR1 signal among 100 interphase nuclei examined.

The patient's treatment course included palliative external beam radiotherapy (EBRT) to the mass at a dose of 20 Gy in 5 fractions that was initiated 17 days after her initial presentation and completed 23 days after her initial presentation. The EBRT resulted in the cessation of her emesis.

The patient also completed 3 cycles of vincristine (2 mg intravenously [IV]), doxorubicin (113 mg IV), and cyclophosphamide (1,800 mg IV) alternating with ifosfamide (2,880 mg IV) and etoposide (160 mg IV) in alternating 14-day cycles initiated 3 weeks after her first presentation and completed 3½ months after her initial presentation. The patient then received treatment with 5 cycles of vincristine (2 mg IV), doxorubicin (113 mg IV), cyclophosphamide (1,800 mg IV), and denosumab (60 mg subcutaneously) beginning approximately 3½ months after her initial presentation and completed 6 months after her initial presentation.

**Figure 2.**
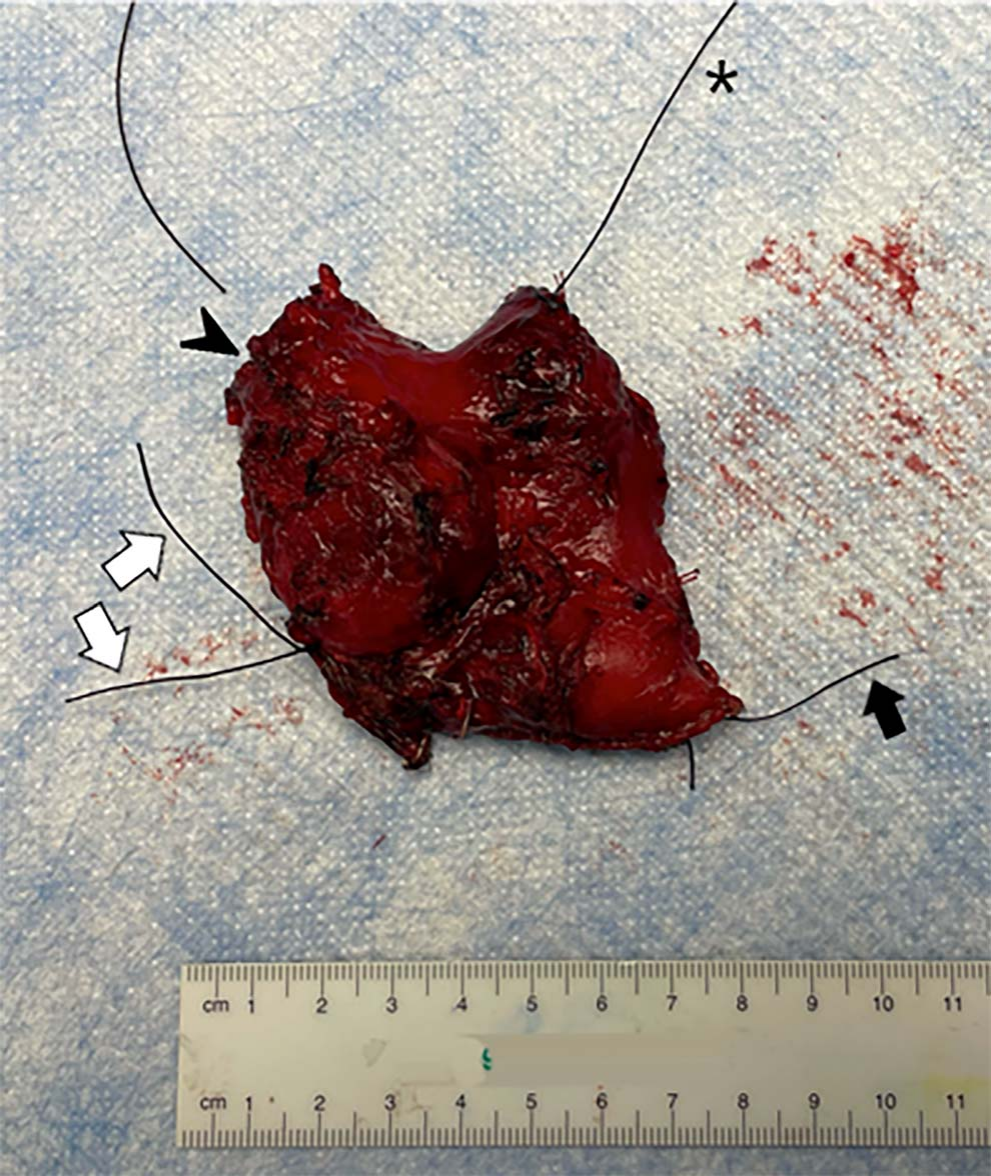
Gross examination demonstrated a 5.2-cm tumor posttreatment (arrowhead) in the right retroperitoneum affixed to the duodenum. The long single suture marks the distal surgical margin (asterisk), and the short single suture marks the proximal surgical margin (black arrow). The double suture on the left represents the tumor margin abutting the superior mesenteric artery (white arrows).

Surgical course after systemic therapy included radical resection of the duodenal mass, open cholecystectomy, wedge gastrectomy, inferior mesenteric artery ligation, and small bowel resection approximately 7 months from initial presentation. The specimen from the radical resection (Figure 2) consisted of a 5.2-cm retroperitoneal mass that was affixed to the duodenum and encroached on the superior mesenteric artery.

**Figure 3.**
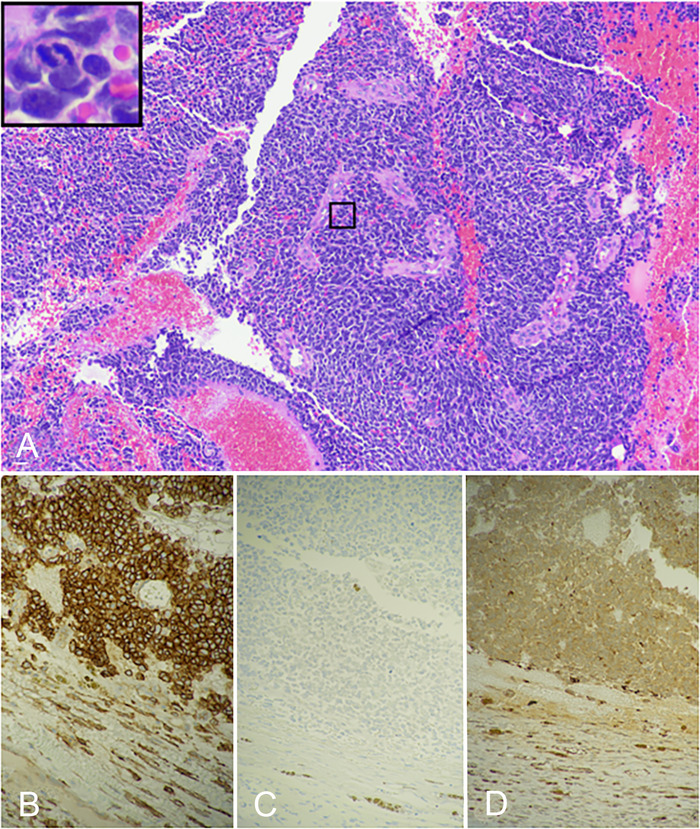
Histologic and immunohistochemistry features of the retroperitoneal tumor. (A) Solid and sheet-like configurations of densely packed tumor cells demonstrate indistinct cell borders, scant cytoplasm, fine chromatin, and inconspicuous nucleoli (hematoxylin and eosin, magnification ×100). The boxed region in the upper left corner shows a tumor cell undergoing mitosis (magnification ×400). The tumor is positive for (B) CD99 and negative for (C) vimentin and (D) WT1 (nonnuclear staining) (magnification ×200).

Cross-sectional analysis of the specimen showed a multiloculated hemorrhagic mass and multiple white-orange plaques within the duodenal wall. Light microscopy showed solid and sheet-like configurations of densely packed tumor cells with indistinct cell borders, scant cytoplasm, fine chromatin, and inconspicuous nucleoli (Figure 3A). The surgical and mesenteric artery margins were clear of tumor cells. Extraskeletal Ewing sarcomas have a tumor differentiation score of 3.^6^ A tumor differentiation score of 3 along with <50% necrosis and 24 mitoses/10 high power fields observed in this case assigns a grade 3 for the tumor (total score 8/9). A single lymph node was identified and was negative for malignancy (0/1). Immunohistochemistry of the resection was positive for CD99 and negative for vimentin and nuclear WT1 expression (Figures 3B, 3C, and 3D). Pathologic staging of ypT2N0 was assigned.^7^

One year from initial presentation, the patient was being actively managed for chronic abscess formation at the surgical resection site. The patient was symptomatically improved and reported 5 to 6 watery bowel movements per day with no other complaints.

## DISCUSSION

ES/PNET is a small round cell sarcomatous tumor that involves the soft tissue and bone. These sarcomas show gene fusions that involve 1 member of the FET family of genes (classically EWSR1) and a member of the ETS transcription factor family. The anatomic distribution of these tumors is wide, and case reports have described manifestations in the kidney, stomach, liver, and small bowel.^8-14^ When involving the small bowel, these tumors usually involve the jejunum or ileum, with involvement of the stomach and duodenum extremely rare. The gastrointestinal manifestations of ES/PNETs are nonspecific. Symptoms of bowel involvement include nausea, vomiting, hematochezia, abdominal pain, coffee-ground emesis, and anemia.^10-12^ To the best of our knowledge, only 3 other cases of duodenal involvement have been described (Table).^11,13,14^

**Table.** Reports of Ewing Sarcoma/Primitive Neuroectodermal Tumors Involving the Duodenum

Study	Age/Sex	Described Location	Unique Imaging Features	Unique Pathologic Features	Size
Adair et al, 2001^13^	21/F	Duodenum and jejunum	Intussusception of the duodenum	N/R	6 × 6 × 4 cm
Kie et al, 2003^11^	20/F	First and second portion of the duodenum	N/R	Invasion of the whole layer of duodenum and focal extension to the pancreatic head	6.5 cm
Huang et al, 2021^14^	41/M	Descending duodenum	Enhancing on CT	N/R	N/R
Present case, 2022	66/F	Transverse duodenum	Heterogenous on CT; intense FDG uptake on PET-CT	Multiloculated hemorrhagic mass	10.3 × 8.8 × 12.3 cm

CT, computed tomography; F, female; FDG, fluorodeoxyglucose; M, male; N/R, not reported; PET, positron emission tomography.

ES/PNETs demonstrate a male to female ratio of 1.4:1.^4^ Most patients are young, and patients >30 years often demonstrate tumor predilection to the soft tissues. While most cases are sporadic, germline mutations have been described. ES/PNETs are associated with FET-ETS fusion genes that encode chimeric transcription factors that regulate numerous genes, and an aberrancy in these transcription factors is thought to lead to the development of these tumors. The most common translocation, present in approximately 85% of cases, is t(11;22)(q24; q12), which results in EWSR1-FL1 fusion transcript and protein.^15^

Grossly, these tumors appear soft and grey-white with areas of necrosis. Histopathologically, ES/PNETs appear as uniform small round cells. These cells typically demonstrate round nuclei, poorly visualized nucleoli, and scant cytoplasm. Chemotherapy can lead to variable degrees of necrosis with replacement by loose connective tissue. CD99 positivity is seen in approximately 95% of ES/PNETs.^16^ NKX2.2 positivity is more specific than CD99.^17^ Keratin expression is seen in 25% of cases.^3^

The radiologic extraskeletal manifestations of ES/PNET are often nonspecific. Therefore, pathologic analysis is required for a definitive diagnosis. On imaging, intra-abdominal ES/PNETs can be misdiagnosed, with imaging features resembling gastrointestinal stromal tumors (GISTs), especially when they are present in the stomach and small bowel. Immunohistochemistry helps distinguish these tumors from GISTs, which are typically positive for CD117, DOG-1, and CD34.^4^ Multimodal therapy for ES/PNETs has improved prognosis considerably. Response to neoadjuvant chemotherapy is a favorable prognostic factor.^18^

## CONCLUSION

This report presents a rare location of the uncommon entity ES/PNET and demonstrates the often difficult and nonspecific clinical presentation of these neoplasms. While ES/PNETs are rare tumors of the abdomen, they should be considered in cases of large soft tissue masses in patients presenting with nonspecific symptoms. To the best of our knowledge, this case is the fourth report in the literature of an ES/PNET involving the duodenum.

## Notes

*This article meets the Accreditation Council for Graduate Medical Education and the American Board of Medical Specialties Maintenance of Certification competencies for Patient Care and Medical Knowledge.*
